# Sustainable Benzoxazine Copolymers with Enhanced Thermal Stability, Flame Resistance, and Dielectric Tunability

**DOI:** 10.3390/polym17152092

**Published:** 2025-07-30

**Authors:** Thirukumaran Periyasamy, Shakila Parveen Asrafali, Jaewoong Lee

**Affiliations:** Department of Fiber System Engineering, Yeungnam University, Gyeongsan 38541, Republic of Korea; thirukumaran@ynu.ac.kr (T.P.); shakilaparveen@yu.ac.kr (S.P.A.)

**Keywords:** polybenzoxazine, biopolymers, copolymer, thermoset polymer, flame resistance, dielectric material

## Abstract

Benzoxazine resins are gaining attention for their impressive thermal stability, low water uptake, and strong mechanical properties. In this work, two new bio-based benzoxazine monomers were developed using renewable arbutin: one combined with 3-(2-aminoethylamino) propyltrimethoxysilane (AB), and the other with furfurylamine (AF). Both were synthesized using a simple Mannich-type reaction and verified through FT-IR and ^1^H-NMR spectroscopy. By blending these monomers in different ratios, copolymers with adjustable thermal, dielectric, and surface characteristics were produced. Thermal analysis showed that the materials had broad processing windows and cured effectively, while thermogravimetric testing confirmed excellent heat resistance—especially in AF-rich blends, which left behind more char. The structural changes obtained during curing process were monitored using FT-IR, and XPS verified the presence of key elements like carbon, oxygen, nitrogen, and silicon. SEM imaging revealed that AB-based materials had smoother surfaces, while AF-based ones were rougher; the copolymers fell in between. Dielectric testing showed that increasing AF content raised both permittivity and loss, and contact angle measurements confirmed that surfaces ranged from water-repellent (AB) to water-attracting (AF). Overall, these biopolymers (AB/AF copolymers) synthesized from arbutin combine environmental sustainability with customizability, making them strong candidates for use in electronics, protective coatings, and flame-resistant composite materials.

## 1. Introduction

Benzoxazines are a relatively new class of thermosetting resins that have gained considerable attention in recent years due to their impressive thermal stability and mechanical strength. When these monomers are cured into polybenzoxazines, the resulting materials exhibit a range of beneficial properties: they resist high temperatures, absorb very little water, experience minimal shrinkage during curing, and maintain excellent mechanical performance. These qualities make polybenzoxazines strong contenders for use in industries like aerospace, automotive, electronics, and advanced composite manufacturing—where materials need to endure harsh conditions without failure. One area where polybenzoxazines truly stand out is their flame resistance. Unlike conventional epoxy resins, which are popular but often flammable, polybenzoxazines inherently offer much better fire safety. For example, commercial diamine-based polybenzoxazine resins, such as poly(DM) (diamine based benzoxazine, synthesized from bisphenol-A, formaldehyde and phenylenediamine), have achieved a V-1 rating in the widely recognized UL-94 vertical burning test. This is already an improvement over many epoxies, and notably, it is achieved without relying on halogen-based flame retardants, which are known to release harmful gases when burned [1,2,3,4,5,6,7,8,9].

Even with these advantages, fire safety in polymers remains a significant concern, especially because burning plastics can release toxic smoke and harmful chemicals. So, the focus in recent research has shifted to making these already flame-resistant materials even safer. The goal is not just to prevent ignition or slow down the spread of fire but also to ensure that any gases released during burning are non-toxic and environmentally friendly. To this end, scientists are exploring both additive and chemical modification techniques to further improve the flame retardancy of polybenzoxazines. Several strategies have been developed to enhance fire resistance. One approach involves adding inorganic flame-retardant fillers. These include materials like aluminum hydroxide, magnesium hydroxide, zirconium phosphate, and nanomaterials such as polyhedral oligomeric silsesquioxanes (POSS). Each of these fillers brings something unique to the table. POSS, for instance, is a hybrid of organic and inorganic structures, known for its stability under heat, resistance to oxidation, and non-toxic nature. It also enhances char formation during combustion, which helps form a protective layer that slows down fire spread. Another route researchers have taken is the use of organophosphorus flame retardants, such as phosphazenes and DOPO (9,10-dihydro-9-oxa-10-phosphaphenanthrene-10-oxide) derivatives. These compounds are widely recognized for their effectiveness in both the gas and solid phases of burning materials. They promote char formation and suppress flame propagation. Because benzoxazine monomers are relatively easy to customize at the molecular level, integrating phosphorus-based flame-retardant groups directly into the structure is quite feasible. This not only enhances fire resistance but also avoids the need for separate flame-retardant additives after polymerization [10,11,12,13,14,15,16,17,18,19].

However, phosphorus-based solutions have recently come under scrutiny. There is growing concern that phosphorus compounds may persist in the environment, especially in soil and water, where they could accumulate over time. As a result, researchers have begun shifting their attention toward more sustainable options, particularly flame retardants that are free of halogens and phosphorus. In this context, bio-based additives and silicon-containing compounds are gaining popularity as greener, yet still effective, alternatives. While improving flame retardancy is critical, another key area of development for polybenzoxazines is enhancing their mechanical performance. In many cases, adding flame-retardant fillers can weaken a material’s structural integrity because of poor dispersion or weak bonding between the fillers and the polymer. This trade-off presents a significant challenge: enhancing fire resistance without adversely affecting the material’s strength, toughness, or flexibility remains a critical objective in material design. To address this, researchers have been developing ways to incorporate flexible segments or nanoscale fillers into the polymer matrix. These modifications help dissipate mechanical stress more efficiently and make the material tougher. For example, Zhao and colleagues created a benzoxazine monomer using polyetheramine, which added flexibility to the resulting polymer. The tensile strength of the cured material was measured at 21.5 MPa. When they added just 3% multi-walled carbon nanotubes (MWCNTs), the tensile strength increased by more than 117%, reaching 46.9 MPa. These nanotubes acted as reinforcement structures, improving the way stress was distributed through the polymer. Another clever technique involves creating dynamic bonds within the polymer network—bonds that can break and reform under stress. These so-called “sacrificial bonds” help absorb energy during impact, improving the material’s toughness. For instance, Wang et al. [3] synthesized polyurethane using isophorone diisocyanate and bisdemethoxy curcumin and blended it with a bisphenol-A-based benzoxazine. The result was a hybrid material (PB-PU) that, once cured, formed a dense network of hydrogen bonds. These reversible bonds dramatically boosted the impact strength by 114%, while also increasing the flexural strength to 147 MPa. However, this flexibility comes with a trade-off. Adding soft, flexible chains or thermoplastic polymers can lower the thermal stability of the material. These components tend to degrade at lower temperatures and can release flammable gases, which undermines the flame-retardant performance. Moreover, their lower char residue limits the protective barrier effect that helps slow fire spread [20,21,22,23,24,25,26,27,28,29,30,31,32,33,34,35,36]. So, the challenge becomes one of fine-tuning these materials to optimize both flame retardancy and mechanical strength. Rao et al. [37] synthesized a compound containing silicon–oxygen (Si–O) bonds and proposed a notable solution to this dilemma. This compound was then reacted with DOPO and p-phenylenediamine to create a new curing agent called DP-PPD. When added to E51 epoxy resin, just 3% of this curing agent was enough for the cured material to reach the highest UL-94 fire rating: V-0. Even more impressively, adding 6% boosted impact strength and flexural strength by 120% and nearly 39%, respectively. The Si–O bonds played a dual role, enhancing mechanical integrity while also contributing to thermal stability and fire resistance. Silicon-based nanomaterials like nano-silica (SiO_2_) have also shown promise in improving flame retardancy. Ran et al. [38] demonstrated that SiO_2_ can react with ammonium polyphosphate (APP) to form silicon pyrophosphate crystals. These crystals reinforce the char layer that forms during burning, acting as a physical barrier that slows heat and mass transfer. The nano-silica also weaves through the char structure, making it denser and more robust. Among all these innovations, POSS continues to stand out as a multifunctional additive. It has proven useful not only for enhancing flame resistance but also for improving the structural properties of polymers. Studies by Yang, Turgut, and others showed that POSS can be used effectively in flame-retardant systems based on APP and pentaerythritol for polypropylene. It forms a hard, ceramic-like layer on the surface when burned, which protects the underlying material. Moreover, POSS can also act as a compatibilizer in polymer blends, helping materials like polyolefins and polyamide 6 (PA6) bond better, especially when POSS has long-chain alkyl groups attached [39,40,41].

Considering the above mentioned facts, this work aims to synthesize two bio-based benzoxazine monomers and their copolymers with improved properties. The integration of arbutin and furfurylamine, which are compounds of biological origin, into the synthesis of the polymeric materials supports their classification as biopolymers. Arbutin, a glycosylated hydroquinone derived from plant sources, and furfurylamine, obtained from agricultural biomass such as hemicellulose-rich residues, serve not only as functional monomeric units but also as renewable feedstocks. Their incorporation reflects a shift toward sustainable material design, aligning with the criteria for biopolymers, which are defined by their partial or complete derivation from biological resources. This bio-based composition enhances the environmental compatibility of the materials and contributes to reducing reliance on fossil-derived monomers.

## 2. Materials and Methods

### 2.1. Materials

[3-(2-aminoethylamino) propyl] trimethoxysilane (AEAPTMS), furfurylamine, and paraformaldehyde were purchased from Sigma-Aldrich (Gangnam, Republic of Korea). Arbutin, dimethyl sulfoxide (DMSO), hydrochloric acid (HCl), chloroform, and methanol were purchased from Duksan Chemicals Co., Ltd. (Ansan, Republic of Korea). All chemicals were used without further purification.

### 2.2. Methods

#### 2.2.1. Synthesis of Bio-Based Arbutin-Derived Benzoxazine (AB) Monomer

The synthesis of the arbutin-based benzoxazine (AB) monomer was carried out using a conventional Mannich-type condensation reaction, utilizing renewable starting materials. In this reaction, arbutin served as the phenol source, AEAPTMS (3-(2-aminoethylamino) propyltrimethoxysilane) provided the amine group, and paraformaldehyde was used to supply the methylene bridge required for forming the oxazine ring. The reaction took place in a 250 mL three-neck flask, fitted with a condenser, thermometer, and dropping funnel. The system was first flushed with nitrogen to maintain an oxygen-free environment. Once the setup was ready, 27.2 g of arbutin (0.1 mol), 6.0 g of paraformaldehyde (0.2 mol), and 22.2 g of AEAPTMS (0.1 mol) were dissolved in 100 mL of dimethyl sulfoxide (DMSO). The mixture was stirred continuously and heated under reflux at 110 °C for 8 h, allowing the reaction to proceed and form the benzoxazine structure through ring closure. After heating, the mixture was left to cool to room temperature, forming a clear, viscous yellow liquid. This product was slowly poured into deionized water to induce precipitation. The resulting solid was washed thoroughly to remove residual impurities and dried under vacuum at 60 °C for 24 h. The final yield of the AB monomer was about 85%.

#### 2.2.2. Synthesis of Bio-Based Arbutin–Furfurylamine Benzoxazine (AF) Monomer

**Scheme 1:**
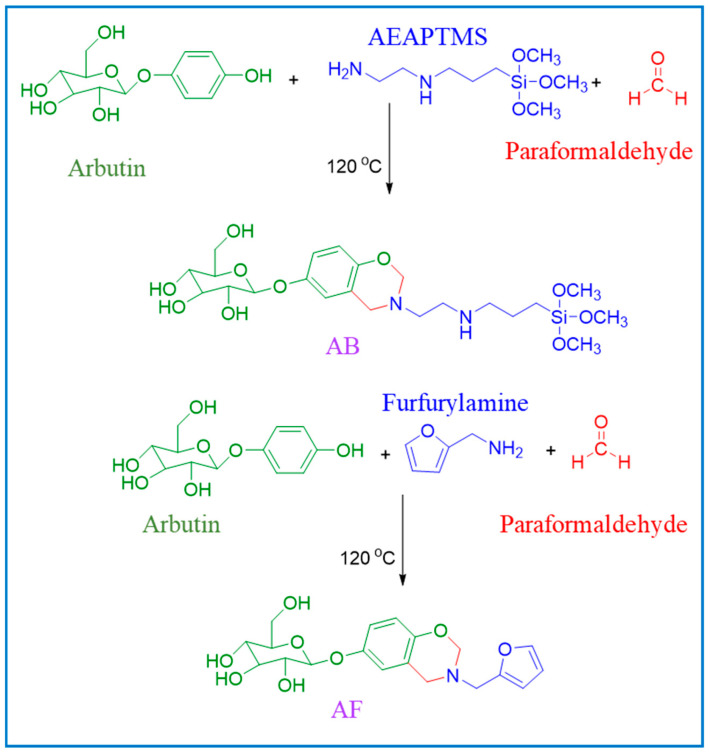
Synthesis of bio-based arbutin-derived benzoxazine monomers (AB and AF).

The synthesis of the arbutin–furfurylamine-based benzoxazine monomer (referred to as AF) was carried out with slight modifications to a method previously reported by Thirukumaran et al. [14], as shown in Scheme 1. To start, arbutin (0.01 mol), paraformaldehyde (0.04 mol), and furfurylamine (0.01 mol) were dissolved in 80 mL of dimethyl sulfoxide (DMSO) in a 250 mL round-bottom flask. The solution was then stirred continuously and heated to 120 °C for six hours, allowing the components to undergo a Mannich-type condensation reaction and form the benzoxazine structure.

Once the reaction was complete, the mixture was cooled to room temperature. It was then filtered to remove any solid impurities. The remaining liquid was concentrated by evaporating the solvent under reduced pressure, resulting in a thick, viscous residue. To isolate the product, this residue was slowly added dropwise to 1N NaOH solution, which caused the monomer to precipitate out of solution. The solid was collected by filtration, washed several times with distilled water to remove any unreacted substances, and then dried under vacuum at 60 °C for 24 h. The process yielded the AF monomer with an efficiency of around 80%. The chemical pathway and structure of the synthesized monomer are presented in Scheme 1.

#### 2.2.3. Copolymerization of AB and AF

To prepare the AB-AF copolymer in a 1:3 weight ratio, 75% AB monomer and 25% AF monomer were measured and dissolved in 10 mL of tetrahydrofuran (THF). The solution was stirred at room temperature until it became completely clear, indicating that the monomers had fully dissolved and blended into a uniform mixture. This homogeneous solution was then carefully poured into a mold and subjected to a staged heat-curing process in an oven. The curing was carried out at four different temperatures, 100 °C, 150 °C, 200 °C, and 250 °C, each held for one hour to gradually promote crosslinking and ensure the monomers were fully polymerized. Following the same method, additional copolymers were synthesized using different AB to AF ratios to observe how changing the monomer composition would affect the properties of the final material. The various weight ratios tested included 100/0, 75/25, 50/50, 25/75, and 0/100 (AB/AF). Each mixture was dissolved in THF, stirred until fully mixed, poured into molds, and thermally cured following the same step-by-step heating schedule. This approach provided a consistent basis for comparing how the different compositions influenced thermal stability and other material characteristics.

### 2.3. Characterization

Fourier transform infrared (FT-IR) spectroscopy was carried out using a Perkin Elmer MB3000 (Hopkinton, MA, USA) instrument in the wavenumber range of 400–4000 cm^−1^ with a resolution of 4 cm^−1^. NMR spectroscopic analysis was conducted on an Agilent AVANCE NEO600 spectrometer (Oxford, UK) at a frequency of 600 MHz to investigate proton environments and other key structural features. Field emission scanning electron microscopy (FESEM) was used to observe the microscopic morphology of the sample. These measurements were taken on a Hitachi S-4800 microscope (Ibaraki, Japan) with an accelerating voltage set at 4 kV. Raman spectroscopy was performed using a Horiba XploRA Micro-Raman system (Palaiseau, France) to analyze vibrational modes over the range of 500–3000 cm^−1^. The wavelength of the laser used is 532 nm, as this directly influences the Raman signal and spectral features. X-ray photoelectron spectroscopy (XPS) was conducted to analyze the surface chemical composition and electronic states, utilizing a Thermo Fisher Scientific K-Alpha spectrometer (Waltham, MA, USA). The radiation source used is Al Kα at 1486.6 eV along with the power settings 12 kV. Other parameters, such as the Gauss–Lorentz ratio 70:30, baseline correction method Shirley, and the fitting procedure for peak analysis are used for XPS spectra decomposition. Spectral deconvolution was performed using CasaXPS software 2.3.26. Thermal behavior was investigated through differential scanning calorimetry (DSC) using a TA Instruments Q200 model (Cleveland, OH, USA). Samples (5−10 mg) were heated under a nitrogen atmosphere at a rate of 10 °C/min. A Dataphysics Instrument OCA 20 model was used to determine the water contact angle of the samples. The measuring range of the instrument was from 0 to 180°. For each sample, contact angle measurement was taken in five different areas, and the average value was denoted. Thermogravimetric analysis (TGA) was performed using a TA Q600 thermal analyzer (Cleveland, OH, USA). Cured samples were analyzed in an open silicon pan at a heating rate of 20 °C min^−1^ in a N_2_ atmosphere, up to a maximum temperature of 800 °C. Dielectric constant and dielectric loss measurements were carried out with the help of an impedance analyzer (Solatron 1260 Impedance/Gain-Phase Analyzer) (Berwyn, IL, USA) at room temperature. The polymer samples were made in the form of pellets (1 mm thickness × 12 mm diameter) using a platinum (Pt) electrode sandwich model in the frequency range of 1 kHz–1 MHz at room temperature. The dielectric constant and dielectric loss of the samples were determined using ε′ and ε″ as the standard relations.

## 3. Results

### 3.1. Structural Confirmation of AB and AF Benzoxazine Monomers

**Figure 1:**
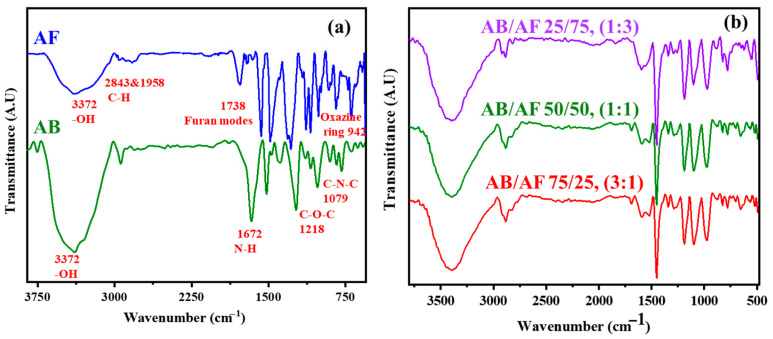
FT-IR spectra of (**a**) AB and AF and (**b**) copolymers.

The successful synthesis of the benzoxazine monomers AB (from arbutin and AEAPTMS) and AF (from arbutin and furfurylamine) was confirmed through FT-IR and ^1^H-NMR spectroscopy. These techniques helped verify the formation of the oxazine ring and the incorporation of key functional groups from their respective starting materials. The FT-IR spectra of both AB and AF (Figure 1a) revealed distinct absorption bands characteristic of benzoxazine structures. For both monomers, the presence of the oxazine ring was confirmed by C–O–C stretching vibrations: asymmetric and symmetric bands appeared at 1218 cm^−1^ and 1042 cm^−1^ for AB, and 1234 cm^−1^ and 1022 cm^−1^ for AF. The C–N–C stretching vibrations, another signature of the oxazine ring, were observed at 1079 cm^−1^ in AB and 1148 cm^−1^ in AF. Both monomers also showed a peak around 942 cm^−1^, which corresponds to the C–H out-of-plane bending of the aromatic ring linked to the oxazine ring. In the AB monomer, a broad absorption at 3372 cm^−1^ indicates the presence of hydroxyl groups from arbutin, while bands at 2843 cm^−1^ and 2958 cm^−1^ in AF reflect alkyl C–H stretching from methylene and methyl groups. AB exhibited additional peaks at 1073 cm^−1^ and 1106 cm^−1^, which are attributed to the vibrations of Si–O and Si-C bonds, confirming the incorporation of the AEAPTMS silane component. On the other hand, AF displayed unique peaks at 1584, 992, and 733 cm^−1^, associated with furan ring vibrations, which support the inclusion of the furfurylamine moiety [39,40,41].

**Figure 2:**
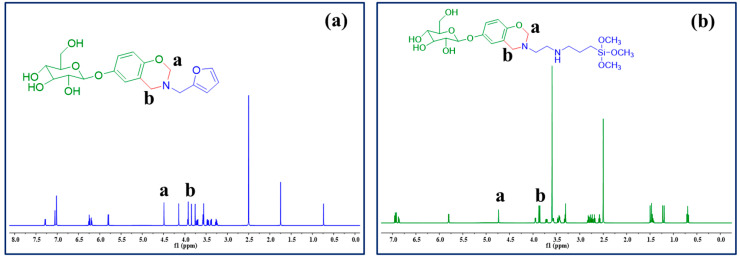
^1^H NMR spectra of (**a**) AB and (**b**) AF.

The ^1^H-NMR spectra (Figure 2) further supported the structural assignments. In both AB and AF, two key singlet peaks confirmed the presence of the oxazine ring: O–CH_2_–N and Ar–CH_2_–N protons were observed at 4.7 ppm and 3.8 ppm in AB, and at 4.5 ppm and 3.8 ppm in AF. These chemical shifts are consistent with the formation of the six-membered benzoxazine ring. For the AB monomer (Figure 2a), multiple signals appeared in the 3.3–5.8 ppm range, which are attributed to the carbohydrate protons of arbutin. A singlet at 3.6 ppm corresponds to the trimethoxy group of the AEAPTMS unit, while additional peaks at 0.7, 1.2, 1.4, 2.7, and 3.4 ppm arise from the alkyl chains and peak at 2.6 ppm arise due to the amine groups of AEAPTMS. In the AF monomer (Figure 2b), the furan ring protons were clearly visible, with signals at 6.2, 6.3, and 7.3 ppm, while aromatic protons showed up as multiplets around 7.1–7.2 ppm. The methylene proton of the arbutin moiety gave singlet at 3.6 ppm, whereas the methyne protons resonate between 6.2 and 6.4 ppm. The peak at 2.5 ppm is due to the DMSO solvent peak. In summary, both FT-IR and ^1^H-NMR results provide strong evidence that the benzoxazine monomers AB and AF were successfully synthesized. The observed signals align well with expected structural features such as oxazine ring formation, presence of aromatic and aliphatic groups, and incorporation of carbohydrate, silane, or furan components—demonstrating the structural integrity of the designed monomers [32,33,34,35,36,37].

### 3.2. Curing Behavior of Benzoxazine Monomers and Their Copolymers

**Figure 3:**
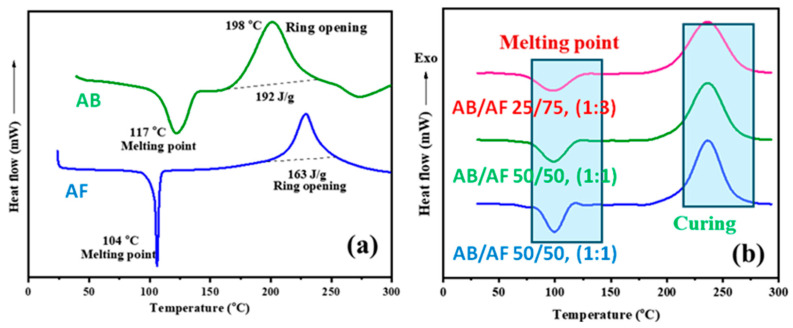
DSC Thermogram of (**a**) AB AND AF monomers and (**b**) copolymers.

The curing properties of the AB and AF benzoxazine monomers, along with their copolymers, were analyzed using differential scanning calorimetry (DSC). These measurements were performed under a nitrogen atmosphere with a heating rate of 10 °C per minute, covering a temperature range from 30 to 300 °C. The resulting thermograms for monomers and copolymers are shown in Figure 3a and Figure 3b, respectively. Table 1 lists the data obtained from the thermograms.

Both monomers exhibited sharp endothermic peaks, which correspond to their melting points and confirm their crystalline nature. The AB monomer melted at approximately 117 °C, while the AF monomer melted slightly earlier at 104 °C. These differences in melting behavior reflect variations in their molecular structures. When AB and AF were mixed in various ratios to form copolymers, each blend showed a single, broadened melting peak, rather than two distinct ones (Figure 3b). This broadening suggests good miscibility between the two monomers and some overlap in their melting transitions. The slight shift to lower temperatures in the copolymers’ melting points is likely due to the earlier melting behavior of AF influencing the blend [42,43,44,45].

The onset of curing, which indicates the start of the ring-opening polymerization process, also varied between the monomers. AB began curing at around 155 °C, while AF started at higher temperature (196 °C). In the case of the copolymers, the curing onset temperatures fell between these two values and gradually decreased as the proportion of AF increased—from about 178 to 174 °C. This shift suggests that AF promotes earlier polymerization, likely due to the electron-donating oxygen in the furan ring, which enhances its reactivity with the benzoxazine ring’s phenolic structure. This trend supports successful copolymerization and interaction between the two monomers. Further into the heating process, exothermic peaks—marking the peak polymerization temperatures—were observed at 246 °C for AB and 255 °C for AF. For the copolymers, this peak appeared in the range of 250–260 °C, consistent with the known behavior of benzoxazine systems, which typically undergo exothermic ring-opening reactions near 250 °C. These findings confirm that both the individual monomers and their copolymers follow typical benzoxazine polymerization pathways.

An important processing parameter derived from DSC is the processing window, which is the temperature range between the melting point and the onset of curing. This window determines how much flexibility is available during processing—before the material starts to cure. AB had a relatively narrow processing window of 38 °C, while AF offered a much wider window of 92 °C, thanks to its lower melting point and higher curing onset. Interestingly, the copolymers also exhibited broad processing windows—averaging around 90 °C—regardless of their specific AB-to-AF ratios. This indicates that the copolymers are not only thermally reactive but also highly processable, allowing sufficient time for molding or shaping before curing begins. In conclusion, the DSC analysis clearly shows that AB and AF monomers undergo ring-opening polymerization typical of benzoxazines. When combined, their copolymers demonstrate tunable thermal behavior, wide processing windows, and excellent thermal workability. These qualities make them attractive for applications that require materials with good thermal stability and flexible processing conditions [31,32,33,34].

### 3.3. FT-IR Analysis of the Curing Process in AB/AF (1:1) Copolymer

**Figure 4:**
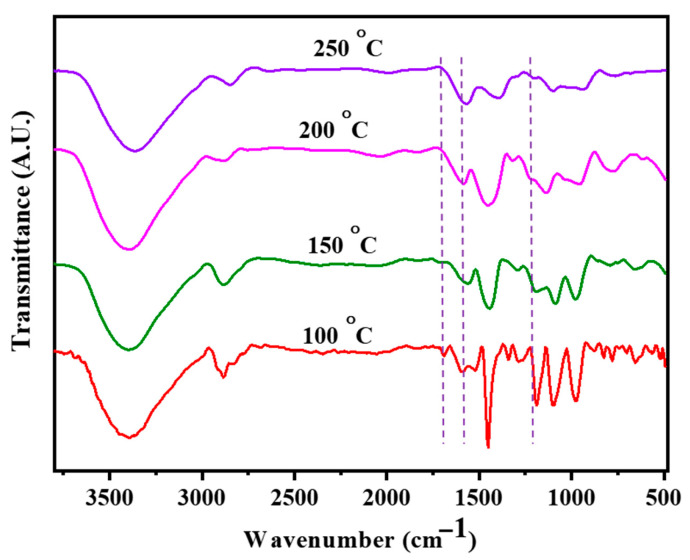
FT-IR spectra of AB/AF 50/50 (1:1) at different analysis temperatures.

**Scheme 2:**
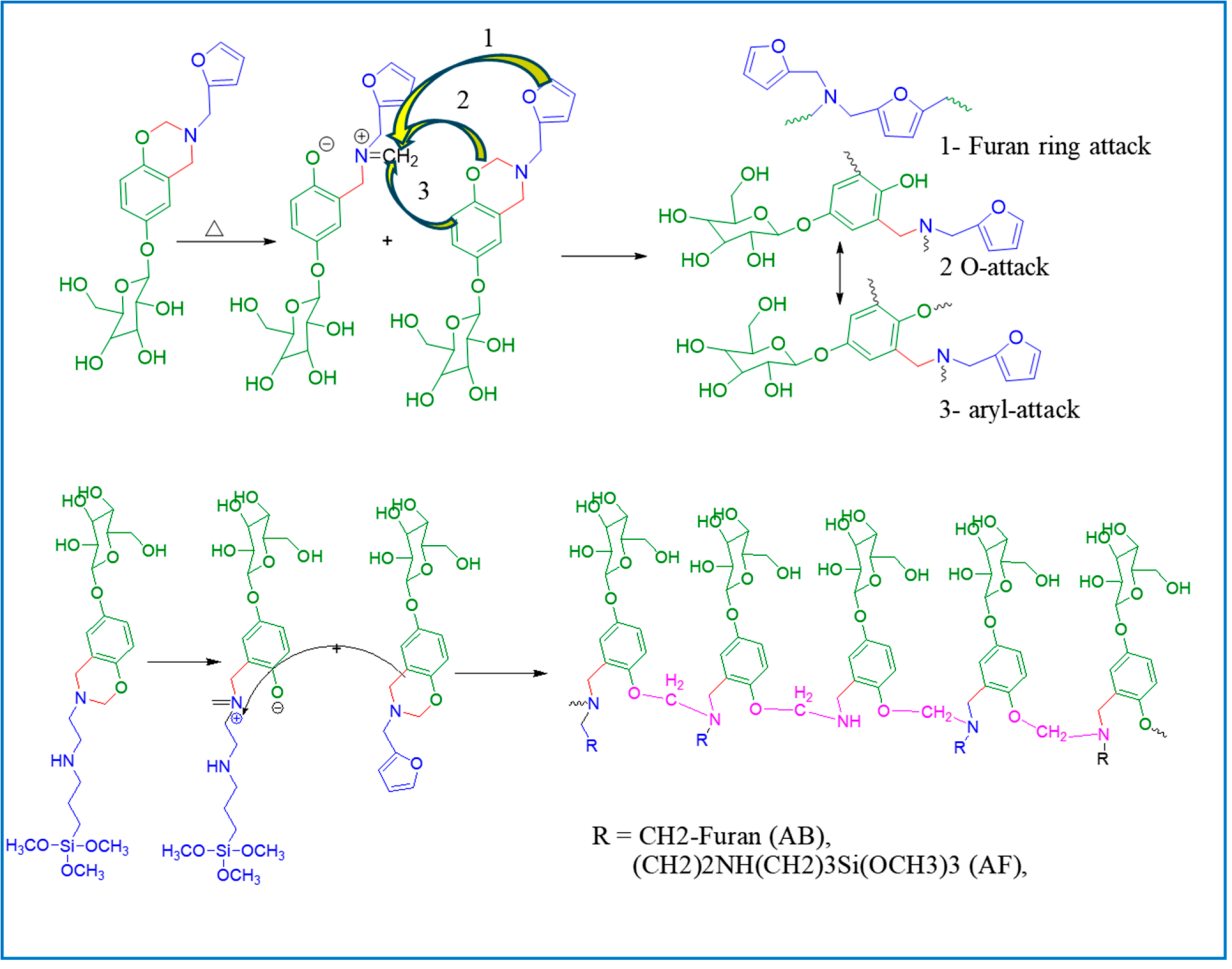
Curing mechanism of benzoxazine monomers and their copolymers.

To better understand the curing behavior of the AB/AF copolymer system, a sample with an equal weight ratio of the two monomers (50:50) was selected for FT-IR analysis at various temperatures. This composition served as a representative model for tracking the structural changes that occur as the material undergoes thermal curing. The FT-IR spectra obtained at different stages of heating are shown in Figure 4. At 150 °C, a decrease in the intensity of key absorption bands—particularly the C–H out-of-plane bending at 943 cm^−1^ and the asymmetric and symmetric C–O–C stretching vibrations at 1227 cm^−1^ and 1034 cm^−1^—indicated the early stages of oxazine ring opening. These changes suggest that the initial breakdown of the ring structure had begun, initiating the polymerization process. As the temperature increased to 200 °C, the intensities of vibrations due to oxazine ring reduced much; meanwhile, the band at 3400 cm^−1^ broadened at these temperatures due to the stretching vibration of phenolic hydroxyl groups. This development confirms that the oxazine rings had opened further, forming hydroxyl functionalities that contribute to the crosslinked structure of the emerging polymer network. By the time the sample reached 250 °C, the characteristic oxazine peaks had almost entirely disappeared from the spectrum, signifying a high degree of polymerization and a nearly complete ring-opening reaction. At this stage, a new absorption band at 1550 cm^−1^ became clearly visible. This peak is attributed to the formation of an alcoholic hydroxyl group, likely resulting from a reaction between the electron-rich furan ring and the ortho-position protons of the phenolic group. The furan moiety, known for its high reactivity due to the presence of an electronegative oxygen atom, facilitates additional crosslinking within the polymer matrix. Together, the spectral evolution observed during heating provides clear evidence of the curing mechanism in the AB/AF copolymer system. The process begins with the thermal ring-opening of the oxazine rings, followed by the formation of phenolic hydroxyl groups, and concludes with crosslinking reactions involving both furan and phenol units. These transformations contribute to the development of a densely crosslinked, thermally stable polymer network. A proposed mechanism for this curing process is illustrated in Scheme 2.

### 3.4. XPS Analysis of AB/AF (1:1) Copolymer

**Figure 5:**
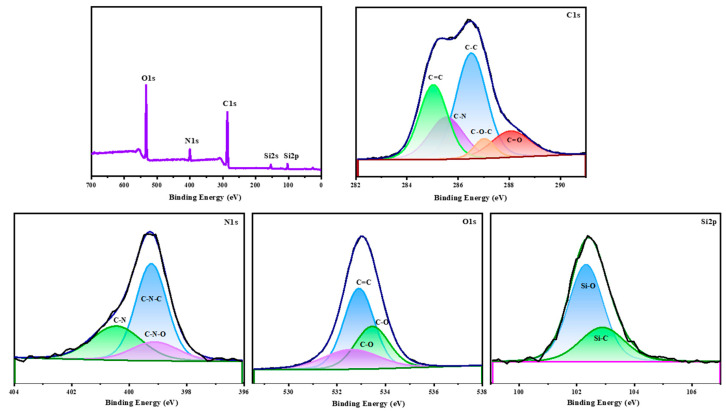
XPS spectra of AB/AF 50/50 (1:1).

To explore the elemental composition and chemical structure of the AB/AF (1:1) copolymer, X-ray photoelectron spectroscopy (XPS) was employed. The overall survey spectrum, shown in Figure 5, revealed the presence of four primary elements: carbon (C), oxygen (O), nitrogen (N), and silicon (Si). These elements are consistent with the chemical components expected from the AB and AF monomer precursors (Table 2). A closer look at the high-resolution C 1s spectrum shows five distinct peaks located at 284.41, 284.67, 285.64, 286.39, and 287.57 eV, each representing different types of carbon bonding. These peaks can be attributed to C=C (aromatic), C–C (alkyl), C–N (amine), C–O–C (ether), and C–OH (carboxy) groups, respectively [36,37,38]. The presence of these diverse carbon environments reflects the chemical complexity introduced during the polymerization process and confirms the formation of various functional groups derived from both monomers. Similarly, the O 1s spectrum was deconvoluted into three main peaks at 532.34 eV, 532.94 eV, and 533.12 eV. The first two correspond to C–O bonds, commonly associated with ether functionality, while the third peak is associated with the hydroxyl functionality, supporting the formation of carbonyl structures in the polymer network [39].

The N 1s spectrum further highlights the diversity of nitrogen-containing groups within the copolymer. Peaks observed at 399.32 eV, 399.26 eV, and 400.54 eV correspond to C–N, C–N–C, and C–N–O bonding environments, suggesting the incorporation of nitrogen atoms from both the oxazine ring and the amine-based components like AEAPTMS and furfurylamine. In addition, the Si 2p spectrum revealed peaks at 101.78 eV and 102.46 eV, which are attributed to C–Si and Si–O bonds, respectively. These peaks confirm the successful integration of silane groups originating from AEAPTMS in the AB monomer structure [44]. Taken together, the XPS data provide strong evidence for the successful synthesis of the AB/AF copolymer. The identification of a range of chemical bonds involving C, O, N, and Si validates the incorporation of structural elements from both monomers and supports the formation of a crosslinked polymer with a chemically rich and well-defined network.

### 3.5. SEM Analysis of AB, AF, and AB/AF (1:1) Copolymers

**Figure 6:**
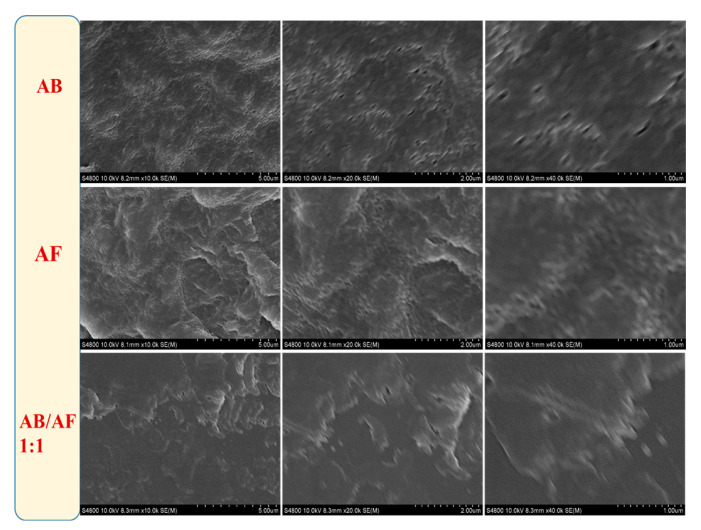
SEM image of AB, AF, and copolymers (1:1).

The surface structures of the AB, AF, and AB/AF (1:1) copolymers were examined using scanning electron microscopy (SEM) at varying different magnifications, as shown in Figure 6. The SEM images of the AB homopolymer reveal a fairly smooth and compact surface with minimal visible imperfections. Even at higher magnifications, only slight textural variations and shallow depressions are noticeable, suggesting a tightly packed and uniform morphology typical of a well-cured thermoset. In contrast, the AF homopolymer displays a much rougher and more irregular surface, with pronounced ridges and layered patterns appearing across the sample. These textural features suggest a looser structural arrangement and possibly increased porosity, likely influenced by the reactive furan rings in the AF monomer, which may lead to a less densely crosslinked network. The AB/AF (1:1) copolymer exhibits a hybrid morphology that reflects features of both individual monomers. The surface appears more structured than that of AF, yet less dense than AB. Moderate surface roughness, small voids, and shallow grooves are evident, indicating effective blending and copolymerization between AB and AF. This balanced morphology suggests good compatibility between the two components, leading to the formation of an integrated and potentially tunable polymer network.

**Figure 7:**
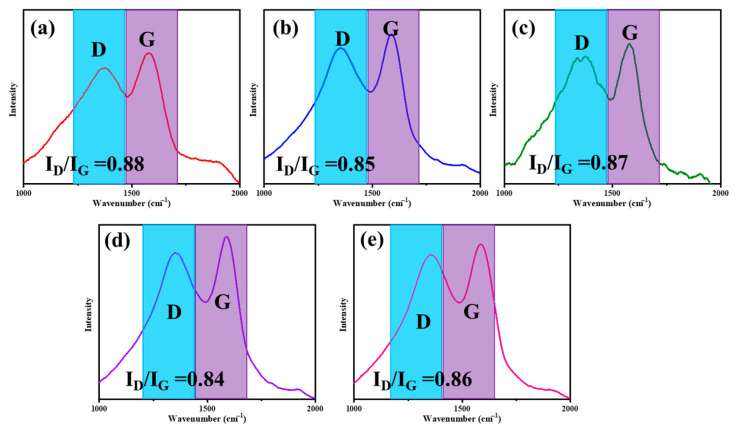
Raman spectra of (**a**) AF; (**b**) AB; (**c**) AB/AF 25/75, (1:3); (**d**) AB/AF 50/50, (1:1); and (**e**) AB/AF 75/25, (3:1).

Raman spectroscopy was also used to further study the graphitization degree of the carbon residue. We can identify in Figure 7 I_D_/I_G_ values of 0.88, 0.85, 0.87, 0.84, and 0.86 for poly(AB) poly(AF), AB/AF 25/75 (1:3), AB/AF 50/50 (1:1), and AB/AF 75/25 (3:1), respectively. The value of AB/AF 50/50 (1:1) is 0.84, which is lower than all the prepared materials, indicating that the residual carbon of AB/AF 50/50 (1:1) has a higher graphitization degree. These results indicate that poly(AB/BF) (1:1) has better solid phase flame-retardancy.

### 3.6. Dielectric Properties of Poly(AB), Poly(AF), and AB/AF Copolymers

**Figure 8:**
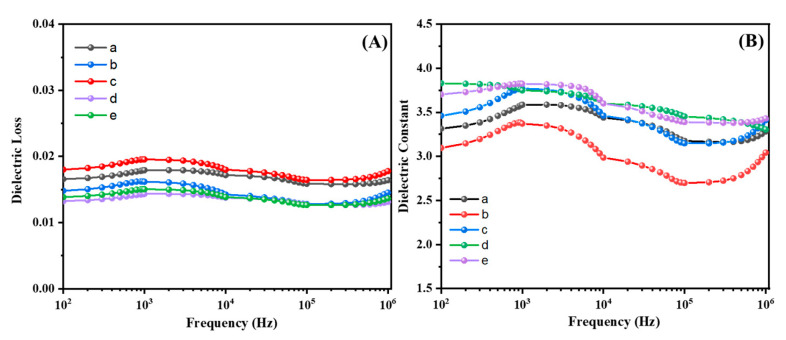
(**A**) The dielectric loss and (**B**) dielectric constant of the synthesized materials (a) poly(AF), (b) poly(AB), (c) poly(AB/AF) (75/25), (d) poly(AB/AF) (50/50), and (e) poly(AB/AF) (25/75).

The dielectric constant (also called the real part of complex permittivity, ε′) measures the material’s ability to store electrical energy via polarization. The imaginary part of the dielectric permittivity, ε′′, represents energy loss due to internal friction, conduction, or lag in polarization. The dielectric properties of poly(AB), poly(AF), and their various copolymers were studied to understand how these materials respond to electric fields, particularly in terms of energy storage (dielectric constant, ε′) and energy dissipation (dielectric loss, tan δ). These measurements, taken across a range of frequencies, offer valuable insight into the molecular dynamics and electrical insulation performance of the materials in Figure 8.

Across all samples, the dielectric constant showed a typical decline with increasing frequency. This behavior is expected in polymers containing polar functional groups, as dipolar polarization cannot keep pace with the rapidly alternating electric field at higher frequencies. Among the homopolymers, poly(AB) consistently had the lowest dielectric constant. This can be linked to the silane-containing AEAPTMS units in the AB monomer, which contribute to a more rigid, less polar environment, limiting the ability of dipoles to align with the field. In contrast, poly(AF) displayed the highest dielectric constant due to the electron-rich furan rings, which enhance dipole alignment and increase polarization efficiency [46,47,48,49,50].

The copolymers, created by blending AB and AF in varying proportions, exhibited dielectric constants that fell between those of the two parent polymers. As the proportion of AF increased, the dielectric constant also rose. For example, the AB/AF (75/25) blend retained a relatively low dielectric constant, closer to that of poly(AB), while the AB/AF (50/50) sample showed a balanced intermediate value. The AB/AF (25/75) composition leaned more towards the high dielectric behavior of poly(AF). This smooth transition in dielectric performance confirms that the electrical characteristics of the copolymer network can be finely adjusted by changing the AB-to-AF ratio.

A similar frequency-dependent trend was observed for dielectric loss. In all cases, tan δ decreased as frequency increased, reflecting reduced dipolar relaxation and energy loss at high frequencies. Poly(AF) again showed the highest dielectric loss, consistent with its more flexible structure and greater dipole mobility, which results in higher energy dissipation. In comparison, poly(AB) demonstrated the lowest dielectric loss, benefiting from a denser crosslinked structure and less polarity, which suppresses dipole motion and enhances its insulating capability.

The copolymers followed the expected pattern: as AF content increased, dielectric loss rose. The AB/AF (75/25) copolymer had a relatively low dielectric loss, similar to poly(AB), while the 50/50 and 25/75 blends showed progressively higher losses, approaching the behavior of poly(AF). These observations suggest that the inclusion of more AF not only increases dipolar polarization but also introduces more flexibility into the polymer chains, allowing for greater movement and, consequently, higher energy dissipation. The opposite trend observed in dielectric constant and dielectric loss is due to the presence of a heterogeneous system, where space charge at the interface enhances the dielectric constant, while reducing the dielectric loss, due to lower conductive pathways.

### 3.7. Thermal Stability Assessment of Poly(AB), Poly(AF), and AB/AF Copolymers

**Figure 9:**
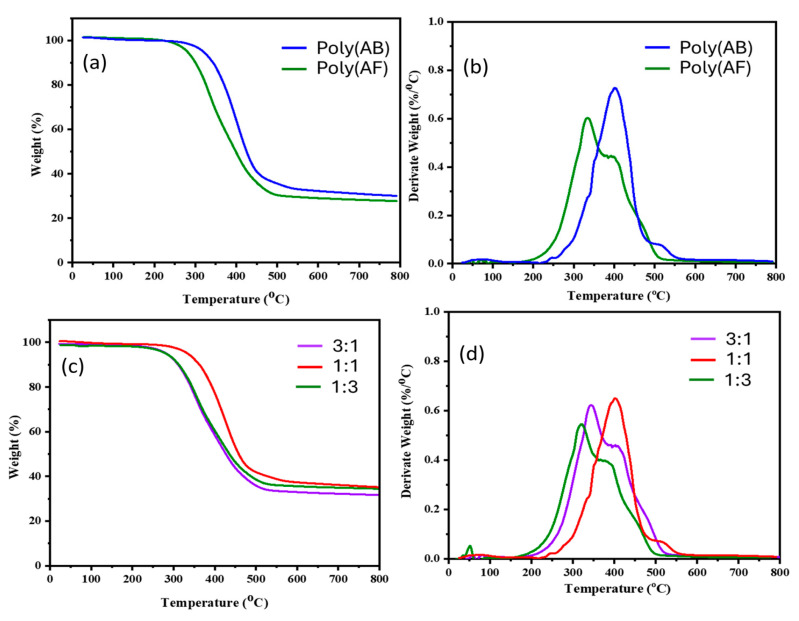
TGA thermogram of poly(AB), poly(AF), and their copolymers (**a**–**d**).

The thermal stability of poly(AF), poly(AB), and their copolymers with varying AB/AF ratios (75/25, 50/50, and 25/75) was evaluated using thermogravimetric analysis (TGA) (Figure 9). The results offer a clear comparison of how each formulation responds to heat and how their structural components influence thermal resistance with data tabulated in Table 3.

Among all the samples, poly(AF) showed higher thermal performance, with degradation temperature slightly higher than poly(AB). The maximum weight loss occurs at 394 and 350 °C for both poly(AF) and poly(AB), as observed from their DTGA graphs (Figure 9b). This is largely due to the presence of the furan ring in its structure, which is known for enhancing thermal robustness. The rigid, aromatic nature of the furan unit, coupled with the stability of the benzoxazine network, helps to delay thermal breakdown. Moreover, poly(AF) produced a relatively high char yield, further confirming its excellent heat resistance and low flammability—features that make it ideal for applications involving prolonged exposure to elevated temperatures [51,52,53,54].

In comparison, poly(AB) began to degrade at a slightly lower temperature. Although it contains silane groups from the AEAPTMS unit, which contribute to some degree of thermal resistance, the overall structure of poly(AB) is less heat-resistant than poly(AF). This reduced stability can be attributed to its lower aromatic content and more flexible molecular structure. Despite this, poly(AB) left behind a significant amount of char after decomposition, which is likely due to the presence of silicon, known to form ceramic-like residues upon heating.

The copolymer with 75% AB and 25% AF showed a moderate improvement over pure poly(AB). The addition of AF’s thermally stable furan ring helped elevate the onset of degradation, and the TGA curve showed a more gradual decomposition, suggesting better interaction between the two monomers. This blend demonstrates how even a modest amount of AF can positively influence the thermal behavior of the copolymer.

At the other end, the 25/75 AB/AF copolymer closely resembled poly(AF) in its thermal profile. It retained the delayed degradation onset and yielded a high amount of residual mass, which points to the dominant effect of AF in the mixture. This composition achieves a good balance between thermal stability and ease of processing, benefiting from both monomer types [51,52,53,54].

The 50/50 copolymer displayed a thermal stability that fell between the two homopolymers. Its degradation temperature and char yield were moderate, reflecting the influence of both AB and AF in roughly equal measure. This balanced formulation offers a middle ground in terms of rigidity, thermal resistance, and material versatility

Overall, the TGA results demonstrate that the thermal performance of these benzoxazine-based polymers can be effectively tailored by adjusting the AB-to-AF ratio. Increasing AF content enhances thermal stability, while a higher proportion of AB contributes to increased ceramic residue upon decomposition. These findings underscore the potential of these materials for use in thermally demanding environments, with tunable properties to suit specific application needs.

### 3.8. Water Contact Angle Analysis of Poly(AB), Poly(AF), and AB/AF Copolymers

**Figure 10:**
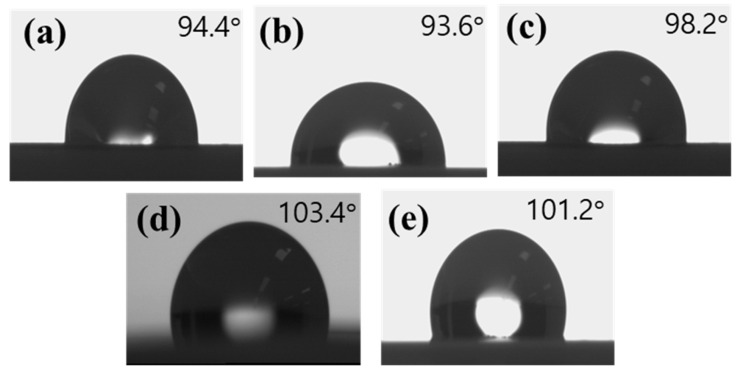
Water contact angle: (**a**) poly(AB); (**b**) poly(AF); (**c**) poly(AB/AF) 25/75 (1:3); (**d**) poly(AB/AF) 50/50 (1:1); (**e**) poly(AB/AF 75/25) (3:1).

Water contact angle measurements were used to assess the surface wettability of the synthesized polymers—poly(AB), poly(AF), and their copolymers with different AB/AF ratios (75/25, 50/50, and 25/75) in Figure 10. This method helps to determine whether the material surfaces are more hydrophobic (water-repelling) or hydrophilic (water-attracting), based on how a water droplet behaves on the surface. Among the materials tested, poly(AB) showed the highest contact angle, suggesting a more hydrophobic surface. This is largely due to the presence of silane groups from the AEAPTMS component, which reduce surface energy and discourage water absorption. These silane functionalities create a tighter, more compact surface structure that naturally repels water.

In contrast, poly(AF) presented the lowest contact angle, indicating a more hydrophilic surface. The furan ring present in the AF monomer introduces polar functional groups that attract water molecules, increasing the material’s affinity for moisture and decreasing the contact angle.

The AB/AF copolymers exhibited contact angle values that fell between those of the two homopolymers. The AB/AF (75/25) sample, which contains more AB, retained a higher contact angle and remained relatively hydrophobic. On the other end, the AB/AF (25/75) sample, rich in AF content, showed a noticeably lower contact angle, indicating increased hydrophilicity. The 50/50 copolymer balanced these properties, displaying a contact angle that reflects an intermediate surface character. These findings clearly demonstrate that the surface wettability of the copolymers can be adjusted by varying the AB and AF monomer content. This tunable hydrophobic–hydrophilic behavior is especially useful for designing materials for specific applications, such as coatings, membranes, or biomedical devices, where interaction with moisture plays a key role in performance.

## 4. Conclusions

This study successfully introduced two bio-based benzoxazine monomers—AB and AF—created from arbutin and functional amines through a straightforward Mannich-type reaction. When these monomers were combined in different proportions, they formed copolymers with a wide range of useful properties. Structural confirmation using FT-IR and ^1^H-NMR confirmed the successful formation of oxazine rings and incorporation of silane (from AB) and furan (from AF) groups. Thermal testing showed that these materials not only cured efficiently but also exhibited high thermal stability. AF-rich copolymers, in particular, resisted degradation better and produced more char, indicating stronger flame resistance. Surface analysis using SEM revealed a shift from dense, smooth textures (AB) to rougher, more porous ones (AF), confirming successful mixing. Electrical testing showed that dielectric properties could be precisely tuned by adjusting the AB/AF ratio. Similarly, water contact angle measurements demonstrated that surface wettability ranged from hydrophobic to hydrophilic depending on composition. These results highlight how easy it is to tailor the properties of these materials simply by changing the monomer mix. Most importantly, since these benzoxazines are bio-based, they offer an environmentally friendly alternative to conventional resins, with great potential for use in electronics, flame-retardant coatings, and other high-performance applications.

## Figures and Tables

**Table 1:** DSC data obtained from DSC thermograms.

Sample	T_m_ (℃)	T_onset_ (℃)	T_max_ (℃)	T_final_ (℃)	PW (℃)
AB	117	162	198	237	45
AF	104	190	227	256	86
AB/AF (75/25)	104	187	238	266	83
AB/AF (50/50)	103	186	240	265	83
AB/AF (25/75)	102	184	242	263	82

T_m_: melting temperature; T_onset_: onset of curing temperature; T_max_: maximum curing temperature; T_final_: final curing temperature; PW: processing window.

**Table 2:** Data showing the theoretical and experimental elemental composition.

Elements	Theoretical (%)			From XPS (%)
	AB	AF	AB/AF (1:1)	AB/AF (1:1)
C	59.4	69.0	63.9	64.7
N	31.3	27.6	29.5	28.9
O	6.3	3.5	4.9	4.6
Si	3.1	0	1.6	1.8

**Table 3:** TGA data obtained from TGA thermograms.

Sample	T_i_ (℃)	T_10_ (℃)	T_50_ (℃)	CY (%)
Poly(AB)	250	302	350	32
Poly(AF)	296	350	394	38
Poly(AB/AF) (75/25)	253	310	356	35
Poly(AB/AF) (50/50)	305	348	396	39
Poly(AB/AF) (25/75)	252	309	354	36

T_i_: initial degradation temperature; T_10_: degradation temperature at 10% weight loss; T_50_: degradation temperature at 50% weight loss; CY: char yield at 800 °C.

## Data Availability

Data are contained within the article.
